# The relative age effect in selection to international team matches in Norwegian handball

**DOI:** 10.1371/journal.pone.0209288

**Published:** 2018-12-19

**Authors:** Christian Thue Bjørndal, Live S. Luteberget, Kevin Till, Simen Holm

**Affiliations:** 1 Department of Coaching and Psychology, Norwegian School of Sport Sciences, Oslo, Norway; 2 Department of Physical Performance, Norwegian School of Sport Sciences, Oslo, Norway; 3 Carnegie School of Sport, Leeds Beckett University, Leeds, United Kingdom; University of North Carolina at Chapel Hill, UNITED STATES

## Abstract

Many elite sport organisations have introduced structured talent identification and development (TID) initiatives in youth sports to better facilitate elite sport performance. However, selection mechanisms for TID programmes (e.g., junior international team) are biased towards relatively older athletes and limited studies exist with Scandinavian contexts. Therefore, the aim of this study was to explore the relative age effect (RAE) in youth, junior and senior male and female international team selections among Norwegian handball players (n = 657). A Chi-square goodness-of-fit test assessed whether a skewed birthdate distribution occurred at the youth, junior and senior international team levels and odds-ratios were calculated for RAE distribution. Moreover, a Kruskal-Wallis test was used to assess differences between the number of international youth, junior and senior level appearances by birth quartiles. Significant uneven birth date distributions were shown for youth (χ^2^(7) = female 40.383 and male 105.716, *p* <0.001) and junior (χ^2^(7) = female 27.427 and male 30.948, *p* <0.001) international players, favouring the relatively older player (odds-ratio of 1.9–8.3). At the senior level, no uneven distribution was identified. The comparison of the number of matches in each age category and the quartile of birth showed a difference in the women’s youth category, where players in quartile 8 had a significantly lower number of appearances compared to quartile 1. The results form part of a growing body of knowledge about selection mechanisms in sport, which favours relatively older athletes within Norwegian Handball. Such findings are important for policy and practice for informing TID programmes for inclusive selection opportunities for all players.

## Introduction

Most elite sport organisations have introduced structured talent identification and development (TID) initiatives in youth sport to better facilitate elite sport performance [1]. Such TID programmes can initiate from as young as 7 years of age, predominantly from 13–14 years, and are often undertaken in chronological (bi)annual-age categories using cut-off dates relevant to the respective nation. For example, in Norway, players born in the same year (i.e., 1^st^ January selection cut off) are selected to the regional player development programme from 13 years of age and onwards. The regional programme is organised in annual-age categories while the national programme starts at age 15 and is organised in bi-annual age-categories. Such a policy decision results in age (dis)advantages based on an individual athletes birth date in comparison to the respective cut-off date [2]. The selection mechanisms of TID programmes are biased towards selecting relatively older athletes (i.e., those born nearer the selection start date) [3].

This well-documented systematic selection bias is known as the relative age effect (RAE) resulting in skewed birthdate distributions among athletes and an over-representation of athletes born closer to age cut-off dates in particular sports [2]. The RAE has been evidenced across all levels of sport, ranging from participation, TID to elite sport across the lifespan [2, 4–6]. Research evidence demonstrates that the RAE is strongest during selection to TID initiatives during adolescence [2] with discrepancies reducing post adolescence in professional adult sport [7–11], although professional football has shown to be an exception [12]. For example, studies have shown, RAEs across selection to formal TID programmes in Swedish football [13]; German football [14, 15]; international football [16]; English Rugby League [17]; Swiss alpine skiing and tennis [18]; German handball [19]; Spanish handball [20, 21]; and international handball [22, 23]. In addition, recently, Bjørndal, Luteberget and Holm [24] investigated the relationship between early and senior level participation in international male and female handball in Norway. It was found that players that participated at the junior international team level are significantly more likely to have played more senior international team matches than players who did not participate at the junior level. This could indicate that RAE during adolescence might affect RAE at senior levels. However, RAEs have not been investigated in Norwegian handball.

Handball is an Olympic, complex and physically demanding team ball sport [25]. It is an intermittent invasion game characterised by repeated high-intensity running and throwing, accelerations and decelerations, powerful change of directions, and full-body contact [26, 27]. Within Norway, handball is predominantly a club-based sport but players may be selected to the regional and national TID programme. From the national TID programme, some athletes are selected to represent the international team at competitions at the youth (Under-18), junior (Under-20) and senior (adult) age categories. International youth and junior handball competitions are organised in bi-annual (2-year) age categories and have been shown to positively influence athlete development in Norwegian handball, and can provide an important arena for high quality practice and competition [28].

Several studies have evaluated the RAE in handball. For example, Schorer, Cobley, Büsch, Bräutigam and Baker [19] found significant RAEs in German male and female handball with increased RAEs at the youth and junior levels compared to senior levels. Furthermore, Gómez-Lopés, Granero-Gallegos, Feu Molina and Chirosa Ríos [20] reported similar RAEs, with 57% of players born in the first half of the year, in young male and female Spanish handball players with no differences between gender or playing position. Similarly, Aguilar, García and Romero [23] noticed a significant age bias, in relation to being born in an even or odd-number year, among all handball players participating in the World Cup at the youth and junior level, between the years 2009 and 2011, but not at the senior level. The bi-annual age categories in international competitions for adolescent players may increase the RAE in selection to international teams.

Although research examining RAEs in handball is available, to the authors knowledge, only a few attempts have been made to investigate the prevalence of RAEs in the context of Scandinavian team sport. Wiium, Lie, Ommundsen and Enksen [29] investigated the presence of RAE among professional football players in Norway. They did not find a linear relationship between the month of birth and membership in a professional club, although they found that more players were born in the first half of the year than the latter. In Danish female handball there was not found any evidence of RAE, rather they found a greater probability of becoming a professional if the player was born in the last quartile (contradicting RAE; Goldschmied, Cobley, Wattie, Baker and McKenna [30]). In selection to international team matches in Danish male handball there was found a significant RAE in the youth and junior age categories for players born between the years 1980 and 1991, favouring the relatively older players [31]. However, more studies are needed in order to more widely consider the impact of RAE across a National TID system within Scandinavia, which could have implications for TID policies within youth and senior sport.

Therefore, the aims of this study were to (a) evaluate the prevalence of the RAE in international youth, junior and senior Norwegian male and female handball players; and (b) explore the relationship between relative age and the number of international youth, junior and senior level appearances. The results should be of particular interest to people developing elite sport policy and practitioners involved in formal TID programmes.

## Methods

### Context

The organisational structure of Norwegian handball includes multiple autonomous actors, such as clubs, sport schools, regional federation-level TID activities, and youth and junior international team activities. In the context of Norway, no actor has instructional authority over others, and no single actor has sole responsibility for talent development.

In both women’s and men’s handball in Norway, the player development model of the National Handball Federation is broad-based: as many as one-third of all youth handball players participate in different TID initiatives during their early adolescence [28]. Athletes are first selected to talent development initiatives at 13+ years. The broad-based talent development initiatives in Norway vary in quality. Players are not officially moved from one level to another and there are no formal threshold measures: it is not unusual for an athlete to return to the talent route after de-selection, and the age at which early specialisation is allowed is partly limited through the formal regulations governing child sport. Although the selections that are made for the Norwegian international handball team can be rotated, monthly five-day training camps are common and many international team players participate in all four of the annual international handball championships throughout their five-year period on their national team. Athletes selected to the youth or junior international teams remain part of their club teams and sport school programmes, and also participate in the regional talent development activities. Studies have shown that the model used is frequently exhausting for many athletes with international team experience and may lead to injuries and burn-out for a significant number of them [32].

**Fig 1:**
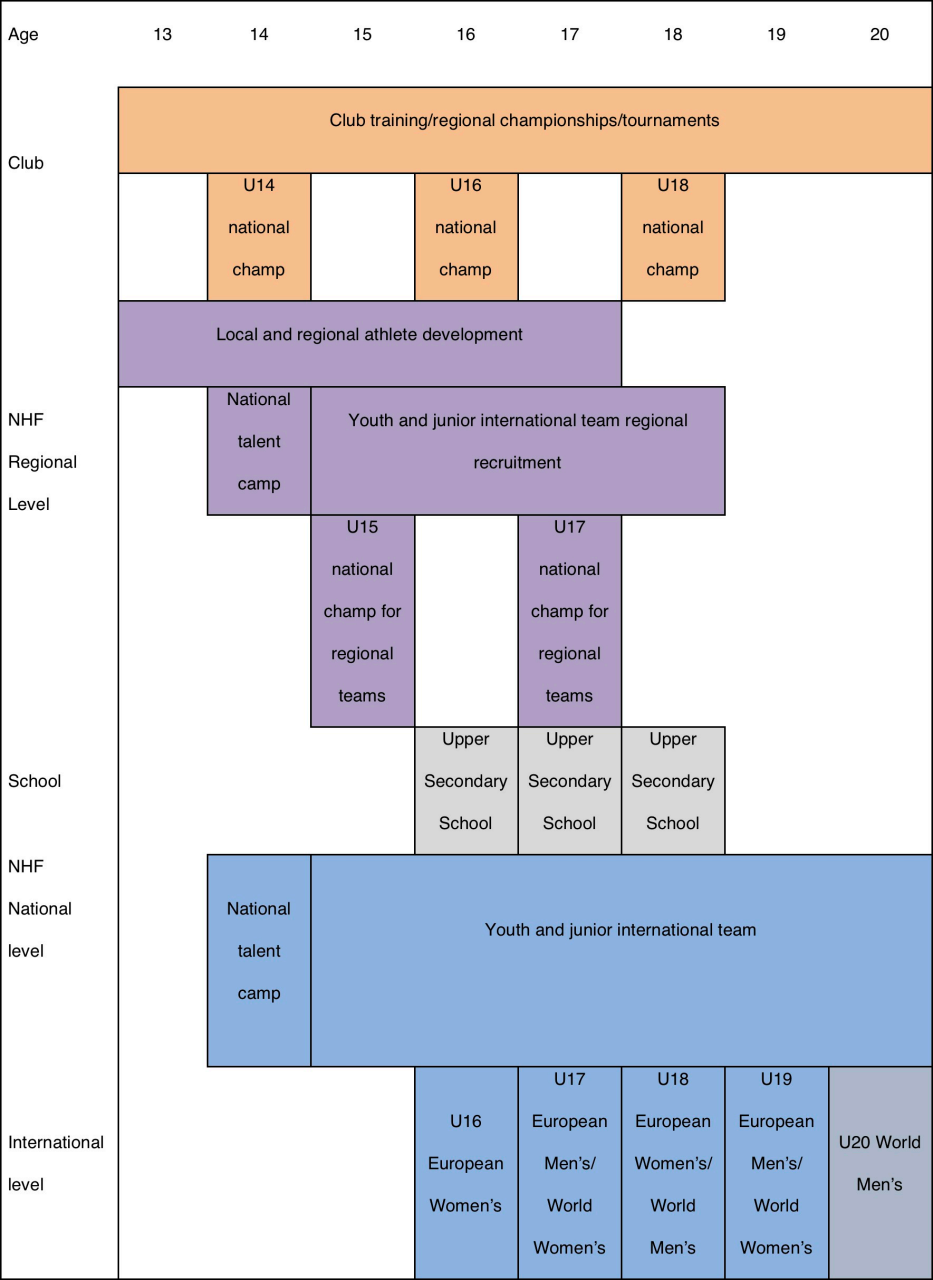
The talent development initiatives in Norwegian youth handball throughout adolescence.

The European Handball Federation and the International Handball Federation each organise, respectively, the European Championships and World Championships once every two years. Both tournaments are played during Europe’s summer holidays and the teams must qualify to take part. Each international team is based on a bi-annual age category: players born in the same two-year period play together throughout their international team careers. The talent development initiatives in Norwegian handball from the age of 15 to 20 years, covers the entire time span for the international youth and junior teams, as shown in Fig 1.

### Participants and procedure

To evaluate the prevalence of the RAE in Norwegian handball a dataset was obtained from the Norwegian handball federation. The study sample consisted of female (n = 318) and male (n = 339) players, born between the years 1986 and 1999, who had been selected to international competitions for the Norwegian international youth (n = 256 and n = 299, for female and male respectively), junior (n = 190 and n = 182, for female and male respectively), and senior (n = 45 and n = 55, for female and male respectively), international teams. Players in the youth category may re-appear in the junior and/or senior categories.

In international handball, all team activities during adolescence are grouped into two-year age categories and those born in the same age-category play together until they enter senior international handball. Within each age category, players who are born in even-numbered years are always relatively older than those born in odd-numbered years. Thus, the study sample consisted of seven different international team cohorts: Players born in 1986–1987; 1988–1989; 1990–1991; 1992–1993; 1994–1995; 1996–1997; and 1998–1999. The international youth team level refers to the under 18 and the international junior team level is equal to the under 20 level.

A list of the team players and their birthdates was obtained with permission from the Norwegian Handball Federation. The list included the number of matches players had played at the youth, junior and/or senior international teams. All the player’s names in this study were anonymised before analysis in compliance with the study’s ethical requirements. Research approval was granted by the Norwegian Centre for Research Data.

Each biannual age-category extended across two years and therefore consisted of eight quarters. Each player’s birthdate was coded into one of these categories (Q1-Q8). In Q1, players were born between the 1^st^ of January and the 31^st^ of March; In Q2, the 1^st^ of April and the 31^st^ of June; In Q3, 1^st^ of July and the 31^st^ of September; and in Q4, 1^st^ of October to the 31^st^ of December, in even-numbered years. In Q5, players were born between the 1^st^ of January and the 31^st^ of March; In Q6, the 1^st^ of April and the 31^st^ of June; In Q7, 1^st^ of July and the 31^st^ of September; and in Q8, 1^st^ of October to the 31^st^ of December, in odd-numbered years. Only Q1 to Q4 was used when evaluating senior players.

### Statistical analyses

The statistical analyses were undertaken using the program IBM SPSS statistics v24. A Chi-square goodness-of-fit test was computed to assess whether there was a skewed birthdate distribution in selection to international team competitive activities in each of the two-year age-categories, in both women’s and men’s handball. Distributions were compared to expected frequencies, set from the official birth statistics of Norway across the years 1988–1999 [33] (Table 1). Odds ratios (ORs) were calculated for RAE distribution in the different international teams: youth, junior and senior, in both women’s and men’s handball respectively. Q1 to Q7 was compared to Q8 for youth and junior, while Q1-Q3 was compared to Q4 for senior players.

To assess possible differences between the quarter of the year in which a player was born and the number of international matches played at the youth, junior and senior international level, a Kruskal-Wallis test was conducted. The significance level was set at p = 0.05. P-values were adjusted by the Bonferroni correction for multiple tests.

**Table 1:** Norwegian birth-distribution according to quartiles, matching the participants in the sample.

Birth year	Q1 (%)	Q2 (%)	Q3 (%)	Q4 (%)	Q5 (%)	Q6 (%)	Q7 (%)	Q8 (%)
**1986–87**	12.3	13.0	12.7	11.3	12.3	13.4	13.0	12.0
**1988–89**	12.6	12.9	12.4	11.4	12.5	13.2	12.9	12.1
**1990–91**	12.9	13.0	12.5	11.7	12.8	13.1	12.6	11.4
**1992–93**	12.8	13.1	12.8	11.4	12.4	13.2	12.7	11.5
**1994–95**	12.5	13.1	12.4	11.9	12.6	13.3	12.9	11.3
**1996–97**	12.6	13.1	12.7	12.0	12.7	13.1	12.7	11.0
**1998–99**	12.4	13.0	13.2	11.1	12.5	13.3	13.1	11.5
**Total**	12.6	13.0	12.7	11.5	12.5	13.2	12.9	11.5

Q = quartile.

## Results

The results show that 61% of all female players and 68% of all male players were born in even-numbered years in selections to the international youth and junior age categories. Table 2 shows the distribution of births for female and male international handball players by each age category and the ORs between each birth quartile. Significant uneven birth date distributions were shown for female youth (χ^2^(7) = 40.383 *p* <0.001) and junior (χ^2^(7) = 27.427, *p* <0.001) international players, favouring the relatively older player. No uneven distribution was identified at the senior level.

**Table 2:** Number of players selected to the different national team categories, with odds ratios.

		Youth		Junior		Senior	
	Quartile	Number of players	Odds ratio	Number of players	Odds ratio	Number of players	Odds ratio
Women	1	38	3.2	31	3.9	13	2.6
	2	52	4.3	39	4.9	13	2.6
	3	51	4.3	33	4.1	14	2.8
	4	26	2.2	13	1.6	5	
	5	31	2.6	24	3.0		
	6	23	1.9	23	2.9		
	7	23	1.9	19	2.4		
	8	12		8			
Men	1	69	7.7	34	2.6	15	1.5
	2	75	8.3	40	3.1	13	1.3
	3	51	5.7	27	2.1	17	1.7
	4	29	3.2	18	1.4	10	
	5	24	2.7	16	1.2		
	6	18	2.0	10	0.8		
	7	24	2.7	24	1.8		
	8	9		13			

For odds ratio Q1-Q7 was compared to Q8 for youth and junior, while Q1-Q3 was compared to Q4 for senior players.

For males, significant uneven birth date distributions were shown for youth (χ^2^(7) = 105.716, *p* <0.001) and junior (χ^2^(7) = 30.948, *p*<0.001) international players, favouring the relatively older players. No uneven distribution was identified at the senior level.

The comparison of the number of matches in each age category and the quartile of birth showed a difference in the women’s youth category (p = 0.007; Table 3). Interestingly, at the senior level, female players in Q4 displayed the highest mean number of matches; however, no significant differences between quartiles was found. No significant differences between quartiles was found for male players.

**Table 3:** Number of matches played per player in the different birth quartiles.

		Youth		Junior		Senior	
	Quartile	Number of matches per player (mean ± SD)		Number of matches per player (mean ± SD)		Number of matches per player (mean ± SD)	
Women	1	25.2	± 16.2	17.3	± 10.8	19.1	± 18.7
	2	18.3	± 14.4	15.7	± 10.8	36.6	± 41.6
	3	17.6	± 15.7	18.8	± 11.8	38.5	± 48.2
	4	14.9	± 13.3	15.8	± 13.2	53.6	± 88.1
	5	14.5	± 13.4	13.9	± 9.6		
	6	15.0	± 13.7	11.1	± 7.2		
	7	13.2	± 13.3	15.4	± 12.2		
	8	6.6	± 6.3 *	15.4	± 13.3		
Men	1	15.7	± 15.1	16.8	± 11.3	34.3	± 42.1
	2	15.2	± 13.0	16.6	± 11.4	32.7	± 23.7
	3	17.1	± 13.7	17.4	± 11.7	23.5	± 26.4
	4	15.1	± 15.2	13.7	± 11.7	24.0	± 34.3
	5	10.6	± 12.5	13.6	± 9.0		
	6	17.0	± 15.0	11.9	± 8.2		
	7	13.9	± 12.9	11.6	± 9.1		
	8	14.7	± 16.9	11.5	± 10.3		

* = P < 0.05 when compared to quartile 1.

## Discussion

Limited studies have examined the RAE in Scandinavian team sports and this study aimed to (a) evaluate the prevalence of the RAE in international youth, junior and senior Norwegian male and female handball players; (b) explore the relationship between relative age and international Handball appearances. Findings showed that relatively older athletes are over-represented in selections to youth and junior international team competitions in Norwegian handball but not at the senior level. These results are consistent with most studies about selection mechanisms in sport showing that there is a clear tendency to select relatively older athletes to formal TID programmes that diminish in adulthood. Such findings may have implications for TID policies in Norwegian male and female handball.

Significant uneven birth date distributions were shown for all female and male players selected to the youth and junior international teams, favouring the relatively older players. Contrary to our findings, some researchers have contended that the RAE is less prevalent in women’s sport [30]. For example, Baker, Schorer, Cobley Bräutigam and Büsch’s [6] study of junior and adult German handball and US soccer found that RAEs were smaller in females even when participation rates were higher, decreased from youth to adulthood, and varied according to playing position. However, the authors of this study based their analysis on first and second league players and did not investigate selections to National talent development programmes or international youth and junior teams. For example, the RAE has been found to be more pronounced among female handball players in the Spanish region of Murcia [34] and in selection to the Spanish Regional Handball Championship for juveniles [20]. Moreover, it has also been clearly demonstrated in the selection to all levels of the talent development system in German handball, including the youth and junior international team level [19]. Therefore, it seems that the lack of RAEs among female athletes previously reported do not prevail in the selection to National TID programmes and international youth and junior competitions in Handball.

Some authors have speculated that the RAE co-varies with the depth of competition in a given sport [6]. The depth of competition in Norwegian handball, as we have argued, is particularly affected by the cultural significance and popularity of handball in Norwegian society [35]. There are currently 85.000 registered youth players under the age of 17 years in Norway, of which two-thirds are female [36]. However, more research is needed to unravel the relative thresholds appropriate to conceptualise the depth of competition in a given sport within a specific context.

In our analysis of the number of matches per player born in the different birth quartiles, only females at the youth international team level (comparison of Q1 and Q8) were found to be different from each other. This may indicate that while international team selections favour relatively older players, they do not strongly affect the possibility of a player achieving a successful international team career after first being selected. These findings echo those of other empirical studies, such as, Bjørndal, Ronglan and Andersen [37] showing that athletes with experience of youth international team activities in Norwegian handball were able to choose from a multitude of potential pathways leading towards the adult elite level. While the 2016 study found that most elite athletes had, on average, more youth international team experience compared with non-elite athletes, more variation was found within the amount of international experience within the elite and near-elite group than between the groups. Similarly, Bjørndal, Luteberget and Holm [24] showed a significant difference between those Norwegian handball players who did or did not have international appearances in the youth and junior age categories, and the number of international appearances at the senior level. However, only non-existent to weak correlations was found between the number of matches played at the international youth, junior and senior team level. In a different but similar fashion, Doyle and Bottomley [38] demonstrated that while there are more players born at the start of the competition year among the 1000 most valuable footballers across the world, their transfer values are no higher, nor are they given more game-time. From a policy-perspective, these studies suggest that the Norwegian Handball Federation should provide more incentives for the initial and continued selection of relatively younger athletes.

In our study, and similar to other studies in competitive handball [19], no uneven distribution between the quarter of birth was observed at the international senior level. While the initial selection to formal talent development programmes is evidently skewed, recent research has suggested that in the transition to senior professional sports, this skewness can be unexpectedly inverted [39]. For example, McCarthy and Collins [40] investigation of selections to and from a professional Rugby Union academy over a 6-year period, found that while late-birth players were less likely to be selected to the academies, the players were more likely to achieve senior professional status. In their 2016 study of academy players in Rugby Union and Cricket over a 10-year period, McCarthy, Collins and Court [8] found evidence of an initial selection bias but, ultimately, a later reversal of the relative age effect advantage. The authors provide a psychologically based explanation of “growth” due to additional challenge experience by these initially disadvantaged younger players but highlight the need for further qualitative investigation to explore this phenomenon in greater depth.

The results from this study highlight how the particular selection to international youth and junior competitions in Norway are favouring relatively older athletes but these findings might also be discussed in relation to how the limited availability of positions is a challenge for all selection-based talent development programmes. This is because the most basic challenge of all talent development programmes that are based on selection is that all criterions for selection are, at best, limited. Most selections are (naturally) based on athletes’ current levels of performance, and for most immature athletes–statistically, the relatively older athletes–the probability of getting selected is greater than for younger athletes. Nevertheless, the fact that athletes are relatively younger does not indicate anything about their potential relative to their older peers. Neither researchers nor practitioners are able to determine athletic potential (through an objective, valid and predictive assessment) and this remains a challenge for selection-based talent development models [41]. Christensen’s [42] study of top-level football coaches clearly shows that talent identification is a socially constructed phenomenon based on the assessments of coaches and experts–assessments that can, occasionally, be subjective. Talent identification should therefore, as Renshaw, Davids, Phillips and Kerhervé [43] suggest, take place at a later stage of an athlete’s development, and closer to his or her peak performance in a given sport.

We are not suggesting that more objective measures of sports talent should be found. The complexity inherent in athlete development would limit the likely success of trying to do so. However, our findings indicate that talent identification has little to do with assessing ‘potential’, and more to do with strengthening the opportunities available to athletes to learn and develop using different material and personal resources [3]. Future research may need to shift beyond the boundaries of documenting the skewed nature of selection mechanisms and focus instead on how systems of athlete development can, even with often very limited resources, support more youth athletes in search of learning, development and performance opportunities. Therefore, policy-makers should not over-utilise selection-based models. Instead, they should encourage the emergence of talent through diverse pathways to elite sport [44]. They should also recognise the importance of making resources available to a more diverse range of athletes and be conscious of the constraints that the RAE may have on young athletes.

Future research should therefore focus more on the mechanisms underpinning talent identification and development. In such, qualitative enquiries may be a particularly promising approach. Moreover, Delorme, Boiché and Raspaud [5] have argued that interpretations in studies of the RAE are potentially limited by the fact that a biased distribution may already be apparent in the population of licensed players. An asymmetry, therefore, would be expected to occur at all selection levels. In this study, we used the birth date distribution of the Norwegian general population between the years 1986–1999, as we were unable to attain the birth date distribution for licensed players within the same period of time in particular. Further investigations of Norwegian handball should attempt to investigate this further.

The results from this study suggest that the selection to Norwegian youth and junior international team competitions is skewed in favour of selecting relatively older players and add to the growing body of evidence that show how static and linear selection methods informing most talent development programmes may be fundamentally problematic. This create unequal opportunities for developing athletes [45]. The effect of doing so may be counterproductive to talent development if the initially selected athletes do not remain at the senior professional level [13].

## Conclusions

Youth handball players’ experiences have revealed that international team experience can be an important developmental influence, and have a strong effect on motivation, skill development and athlete learning [46]. Youth and junior international team activities and competitions could therefore be systematically rotated to minimise the risks of injuries or overuse, to maximise the potential learning outcomes for athletes *and* minimise selection bias.

In handball, two possible strategies could help to counteract the relative age effect. Firstly, coaches involved in talent development programmes should be actively aware of the relative age effects that occur during selections, such as those for youth and junior international team competitions. Secondly, as Aguilar, García and Romero [23] have proposed, a change is needed to the age categories in youth and junior international competitions, such that the older players the group should be allowed to change category each year. The former strategy may help to reduce the prevalence of the relative age effect; the latter might help decrease the tendency to choose players born in even-numbered years. These strategies may help create more equal opportunities for handball players.

## Supporting information

S1 File Data set. (CSV)
